# Supplementary material for: The Sin3A/MAD1 Complex, through Its PAH2 Domain, Acts as a Second Repressor of Retinoic Acid Receptor Beta Expression in Breast Cancer Cells
Source: Cells. 2022 Mar 31;11(7):1179. doi: 10.3390/cells11071179 (PMC8997856; doi:10.3390/cells11071179)
Supplement: Supplementary file 1 [file cells-11-01179-s001.zip › cells-1566242-supplementary.pdf]

Supplementary Figures

Fig S1

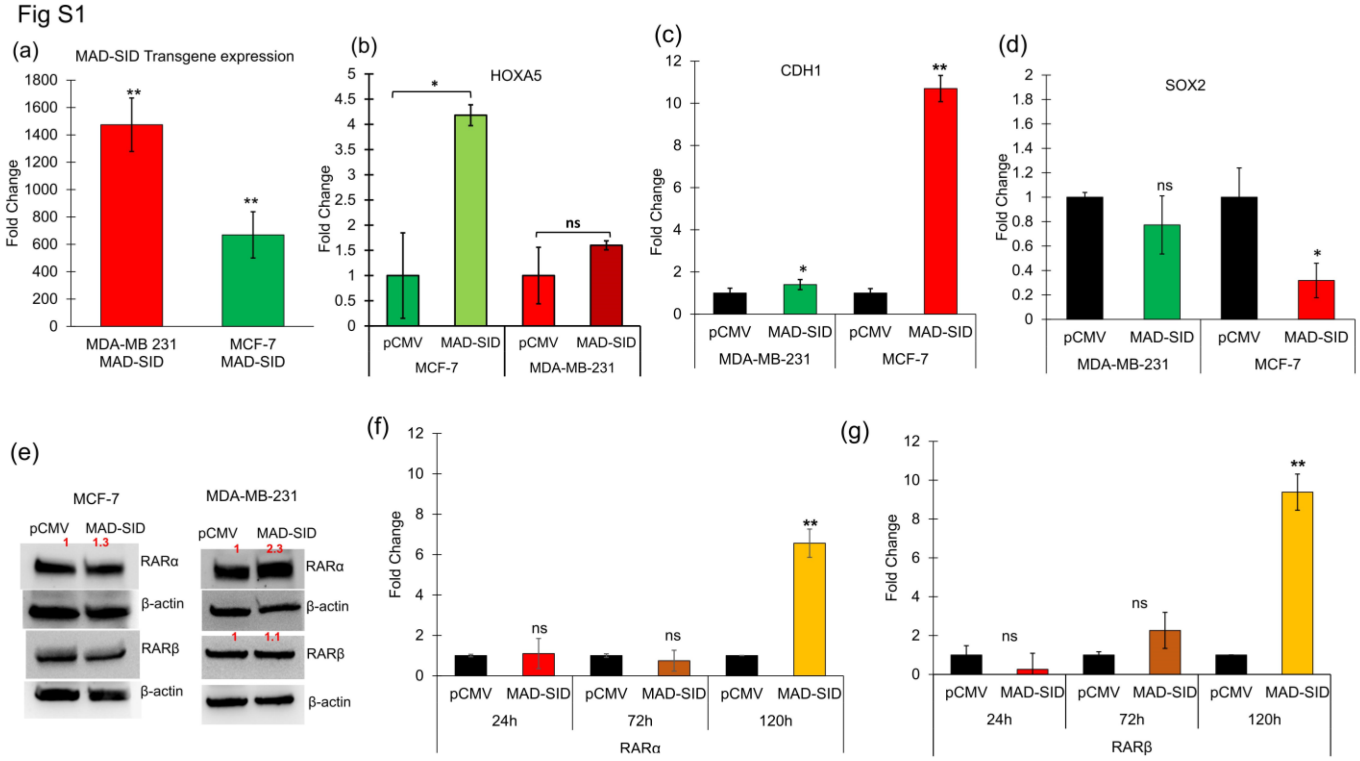

Figure S1. Expression of MAD-SID in MDA-MB 231 and MCF-7 cells increases expression of HOXA5 and differentiation markers: a) MDA-MB-231 and MCF-7 cells were transfected with pCMV and MAD-SID. The expression of transgene was estimated through RT-PCR. The results are expressed as increased fold change expression of transgene in MAD-SID transfected cells as compared with pCMV obtained from a minimum of three independent replicate experiments. b)pCMV and MAD-SID expressing MCF-7 and MDA-MB-231 cells were analyzed for the expression of HOXA5 which is an RARβ gene. RTPCR results show an increase in HOXA5 in both MCF-7 and MDA-MB-231 cells. c) & d) The expression of CDH1 and SOX2 was estiomated through RT-PCR. e) Whole cell lysates of pCMV and MAD-SID overexpressing MDA-MB-231 and MCF-7 cells were subjected to Western blotting and visualized through chemiluminescence. The results presented are representative of average fold change obtained from two independent experiments. f) and g) The expression of RARα and RARβ was estimated at successive time-points in cells expressing MAD-SID and the control vector. The data presented are from two independent experiments. Statistical analysis was conducted using unpaired ‘*t*’ test, error bars represent mean±SD. * $p < 0.05$, **$p<0.01$, ns $p>0.05$.
